# Supplementary material for: Exploring the anti-aging effects of fisetin in telomerase-deficient progeria mouse model
Source: PeerJ. 2023 Dec 12;11:e16463. doi: 10.7717/peerj.16463 (PMC10722989; doi:10.7717/peerj.16463)
Supplement: Supplemental Information 1 [file peerj-11-16463-s001.docx]

**pAkt**





**Akt**





**Bcl-2**





**β-tubulin**
