# Supplementary figures and images for: Exploring the anti-aging effects of fisetin in telomerase-deficient progeria mouse model
Source: PeerJ. 2023 Dec 12;11:e16463. doi: 10.7717/peerj.16463 (PMC10722989; doi:10.7717/peerj.16463)

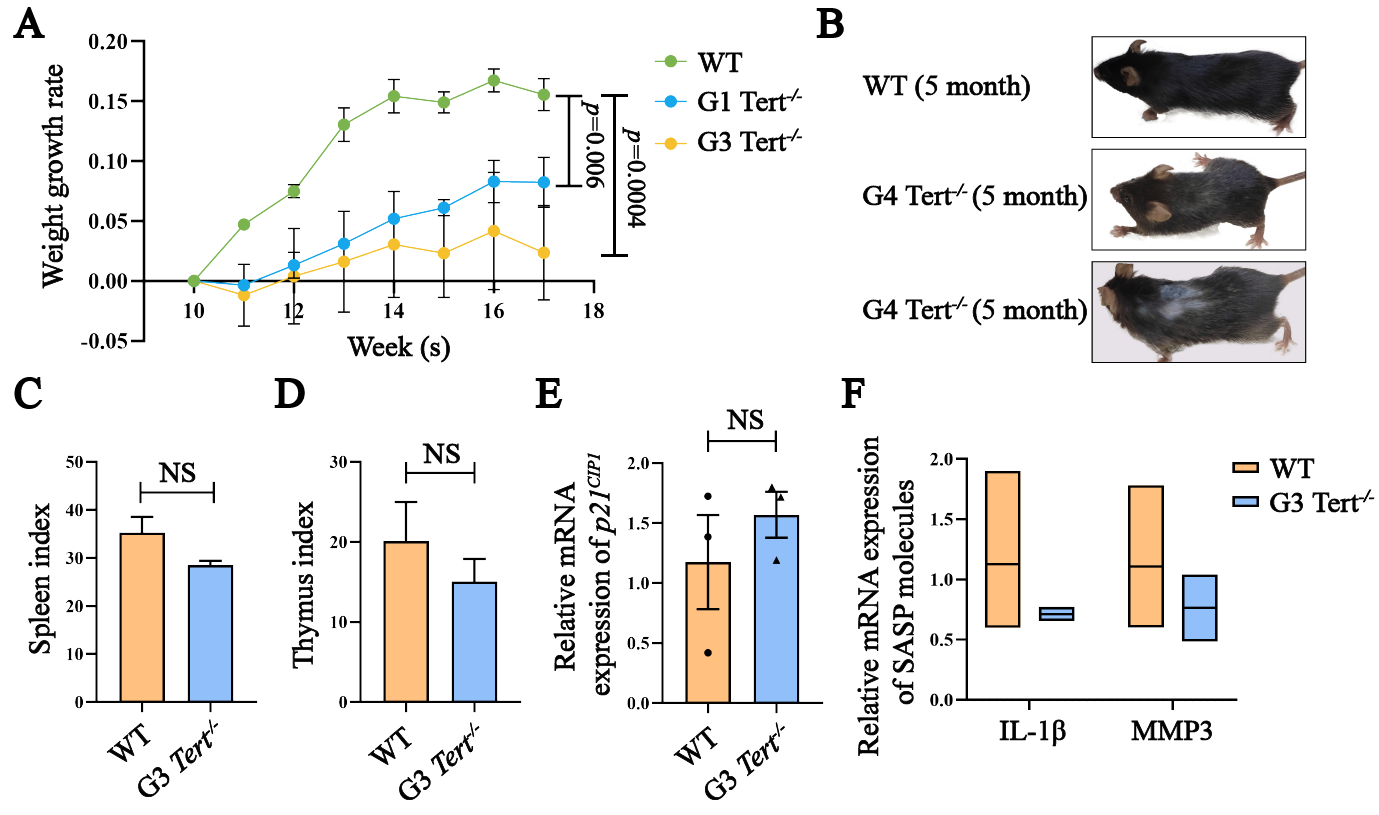

Supplement: Supplemental Information 3 — (A) Weight growth rate of WT (n = 5), G1 Tert−/− (n = 5), and G3 Tert−/− (n = 4) mice. The mice were weighed starting at ten weeks of age, with weekly measurements conducted for a total of seven weeks. (B) WT and G4 Tert−/− mice are representative examples of coarseness , gray, and hair loss in fur. (C–D) Spleen and thymus index of 17-week-old WT (n = 5) and G3 Tert−/− (n = 4) mice. (E) The relative mRNA expression of p21CIP1 in the lungs of 17-week-old WT (n = 3) and G3 Tert−/− (n = 4) mice. (F) The relative mRNA expression of SASP molecules (IL-1 β and MMP3) in spleen of 17-week-old WT (n = 3) and G3 Tert−/− (n = 2) mice. Data represents the mean ±SEM using unpaired t-test. [file peerj-11-16463-s003.png]

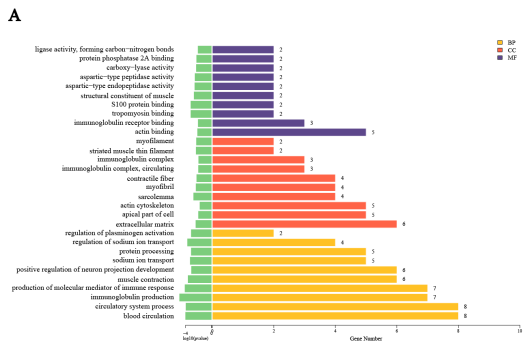

Supplement: Supplemental Information 4 — A. Gene Ontology (GO) biology analysis displaying the enrichment status of pathways for the 83 upregulated differential genes in the G3 Tert−/− + Vehicle group. [file peerj-11-16463-s004.png]
